# Perioperative Opioid Usage Monitoring and Waste

**DOI:** 10.31480/2330-4871/185

**Published:** 2024-05-07

**Authors:** Renyu Liu, John Grothusen, Scott Falk

**Affiliations:** Department of Anesthesiology and Critical Care, Perelman School of Medicine at the University of Pennsylvania, USA

**Keywords:** Opioid, Perioperative, Waste, Diversion, Monitoring, Detection

## Abstract

This editorial discusses the status and issues related to perioperative opioid usage monitoring and waste. Opioid detection of wasted material is briefly discussed also. Flowlytics^®^ from Invistics is a digital system to monitor opioid usage and waste in medical facilities. Opioid waste in medical facilities has a two-person witness procedure. Easy to use detection of wasted materials needs to be developed in the future. It is unclear whether the strategies used in medical facilities should be recommended for opioid disposal in the public to reduce opioid diversion. Relevant studies are needed.

## Introduction

The perioperative period is one of the places in a hospital where opioids are used on a daily basis. Areas where opioid diversion can occur in the hospital include the perioperative area where much of the diversion might go undetected. An early study published in 1987 in The Journal of the American Medical Association indicated that general practice, family medicine, and anesthesia are practice groups on the top of the list with high risk of substance dependence [1]. Anesthesia providers are more likely to have opioid use disorder (OUD) than other sub-specialties, with most of the abuse occurring through the intravenous route [1]. Anesthesiologists constitute 13–15% of people receiving substance dependence treatment in centers specialized in substance dependence management and monitoring for physicians [2]. One of the cited reasons why anesthesia providers have a higher incidence of OUD is their easy access to such medications [3]. Opioid usage for patients in hospital settings is generally very tightly controlled and monitored. Disposal of opioid waste in the perioperative area requires a two-person witness procedure, to ensure there are no discrepancies in the amount used in patients and the amount to be discarded. Disposal of opioids was less regulated in the past with unused opioids being dumped into regular trash or into a sink. Many practices now use a specific process bin or container to discard opioid medication. Opioid usage for patients after surgery is generally managed by the surgical team. To improve the safety of opioid usage and deter diversion in the perioperative period, innovative initiatives and strategies need to be developed, studied and implemented.

## Diversion Monitoring and Detection

**Figure 1:**
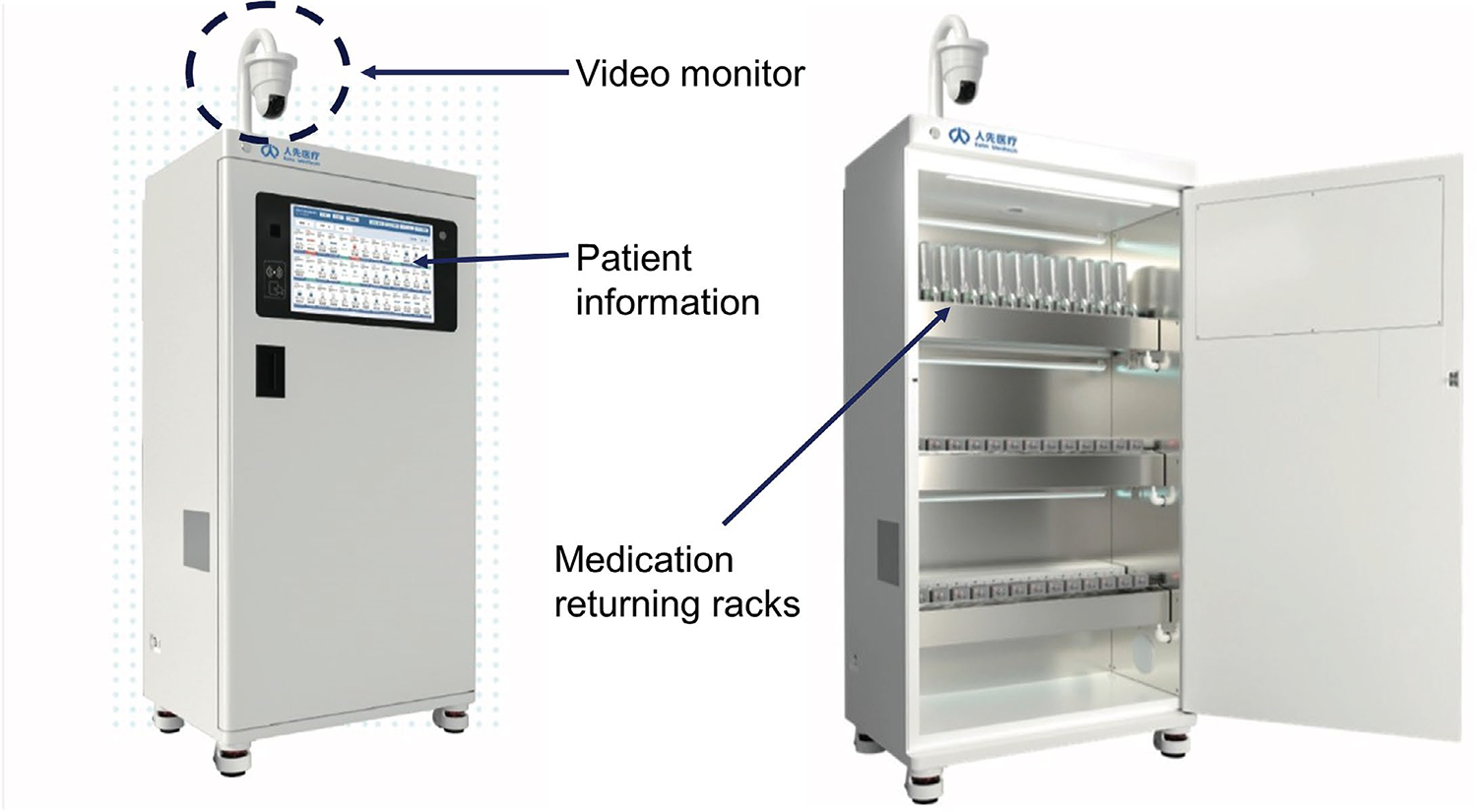
The patient controlled analgesia medication unused medication returning system developed by Rehn Meditech (Courtesy of Rehn Meditech).

Flowlytics^®^ from Invistics was developed using artificial intelligence (AI) to monitor opioid usage and to detect potential diversion. A recent study indicated that use of machine learning and advanced analytics can detect known diversion cases much faster (ranges from 7–579 days faster) than traditional diversion detection methods that use periodic reporting [4]. This new methodology has demonstrated more than 95% accuracy, specificity, and sensitivity, and is designed to alert investigators for potential drug diversion by quickly detecting: 1) **Lack of or discrepancy in** drug reconciliation, 2) **Time-range matrix** to detect significant differences in drug waste behavior from a particular provider as compared to peers in similar cases and 3) A pattern of **full dose wasting** where a full vial of unused medication goes into waste instead of being returned [5]. The Flowlytics software has been part of the Sentri 7 Suite from Wolters Kluwer since June 2023. It is a requirement now that opioid waste must be witnessed by a second person. In China, the opioid waste process is often video recorded, and all opioid ampoules must be returned to the pharmacy and documented. Figure 1 shows the artificial intelligence (AI) driven opioid waste and monitoring system developed by Rehn Meditech, China, for its patient controlled analgesia system, that ensures that opioid containing solutions are discarded properly and recorded. A recent study using hospital registries indicated that use of smaller syringes could reduce perioperative waste, cost and perioperative adverse effects [6]. Further research is needed to demonstrate whether such monitoring or data registry will reduce opioid diversion in the hospital settings.

## Opioid Disposal in Perioperative Area

**Figure 2:**
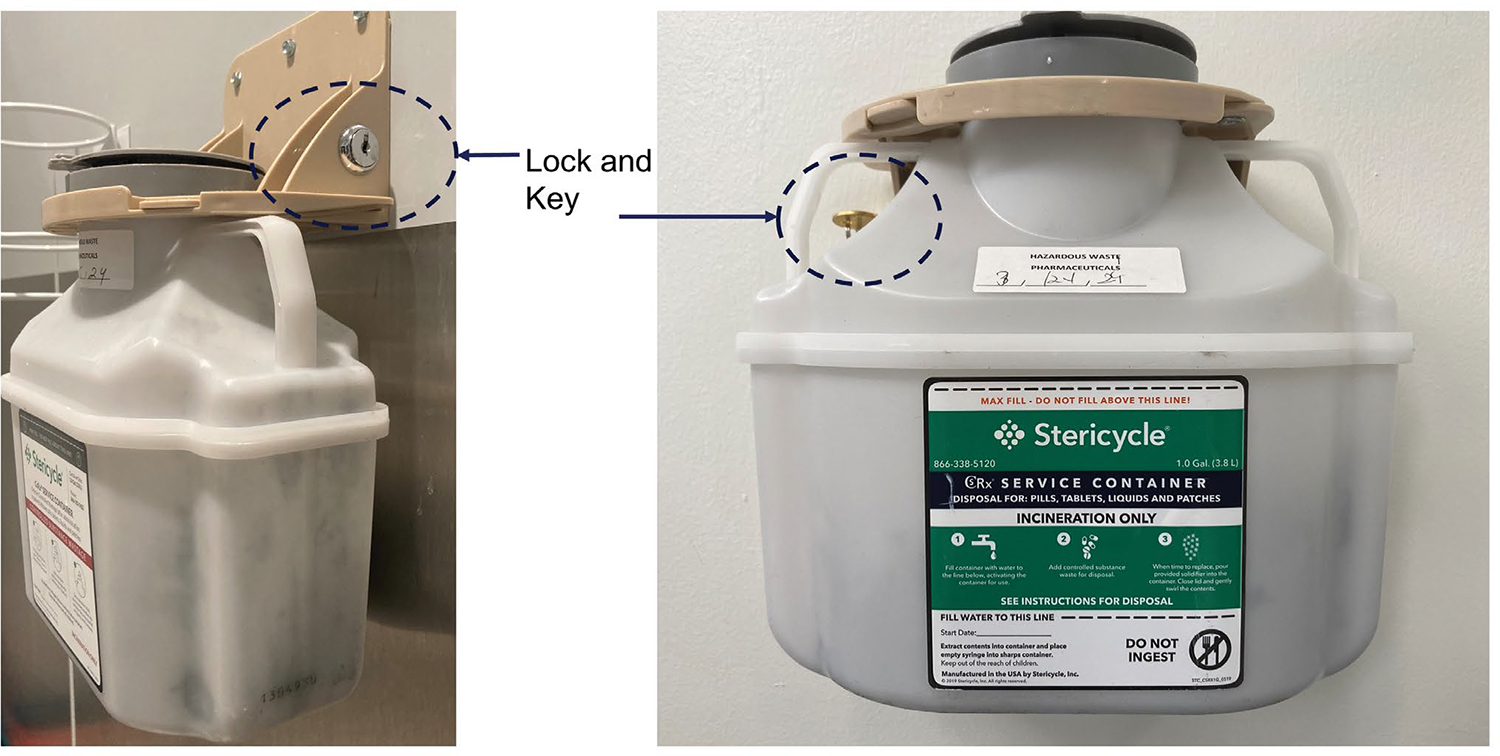
Opioid disposal container from Stericycle.

Opioids are the most commonly used pain medications in the perioperative period. Previously it was routine practice that unused opioids were dumped into trash bins or into a sink. Most opioids are, in fact, on the safe flush list of the USA Food and Drug Administration (FDA) [7]. In a study published in 2017, where 15 active ingredients were investigated for potential environmental impact, it was concluded that most of the investigated drugs flushed down the toilet do not pose significant environmental risk. However, the authors did claim that additional data are needed [7]. With the increasing consumption of opioids, and the current opioid crisis, there is a growing concern of discarded opioids getting into the environment and generating negative impact on human and animal health. The guidelines on how to dispose opioids safely have been updated in multiple safety agencies. The last update on the FDA website for “safe disposal of medicines” was November 2021 [8]. A recent study indicated that trace amounts of tramadol, a relatively weak opioid, in water could change fish behavior. Studies analyzing potent opioids, including morphine and heroin, in the wastewater of two USA cities with known drug overdose problems indicated that the number of overdose deaths correlated with the concentration of opioids detected in the wastewater [9]. To reduce the release of opioids into the environment, and deter potential diversion, new products for opioid disposal have been developed. Many institutions have adopted a policy that all unused opioids must be disposed of in a dedicated deactivation container. An example of a deactivation container from Stericycle is shown in Figure 2. The controlled substance is deactivated with activated charcoal with a bittering additive and solidifier added to further deter diversion.

## Opioid Detection

Opioid detection is critical for not only patient and medical safety, but for environmental safety monitoring and national security. An ideal opioid detection system must have high sensitivity and specificity, and be easy to use with portability and a fast detection process that can be used in various field situations. Fentanyl strips are available now and are easy to use; however, they suffer sensitivity issues and false positive problems [10]. By using opioid receptor protein and graphene enabled technology, we and others have demonstrated high sensitivity in detecting opioids [11–13]. Such opioid protein based technology may ultimately prove to be the most sensitive and specific way to detect opioids and metabolites in the environment [12–15]. However, portability of such technology is limited at the present time.

In summary, this paper discusses the status of perioperative opioid monitoring and waste strategies to reduce potential diversion and OUD. It is unclear whether some of the strategies (for example, two-person witness procedure for opioid waste) will be suitable and adaptable for general public and household opioid management to reduce diversion and help to abate the OUD crisis. This topic is beyond the focus of this editorial. Further discussion and studies are needed before any new policy or guideline can be recommended.
